# Adoption in Eastern Grey Kangaroos: A Consequence of Misdirected Care?

**DOI:** 10.1371/journal.pone.0125182

**Published:** 2015-05-13

**Authors:** Wendy J. King, David M. Forsyth, Graeme Coulson, Marco Festa-Bianchet

**Affiliations:** 1 School of Biological Sciences, University of Queensland, St. Lucia, Queensland, Australia; 2 School of BioSciences, University of Melbourne, Parkville, Victoria, Australia; 3 Département de biologie, Université de Sherbrooke, Sherbrooke, Québec, Canada; University of Sydney, AUSTRALIA

## Abstract

Adoption is rare in animals and is usually attributed to kin selection. In a 6-year study of eastern grey kangaroos (*Macropus giganteus*), 11 of 326 juveniles were adopted. We detected eight adoptions by observing behavioural associations and nursing between marked mothers and young and three more by analysing the relatedness of mothers and young using microsatellite DNA. Four adoptions involved reciprocal switches and three were by mothers whose own pouch young were known to subsequently disappear. Adoptive mothers were not closely related to each other or to adoptees but adoptive mothers and young associated as closely as did biological pairs, as measured by half-weight indices. Switch mothers did not associate closely. Maternal age and body condition did not influence the likelihood of adoption but females were more likely to adopt in years with high densities of females with large pouch young. Adoption did not improve juvenile survival. We conclude that adoptions in this wild population were potentially costly and likely caused by misdirected care, suggesting that eastern grey kangaroos may have poorly developed mother-offspring recognition mechanisms.

## Introduction

Adoption, the exclusive care of conspecific non-offspring, is rare in animals [1]. Instances of alloparenting, including nursing, food provisioning, guarding, carrying and grooming, however, have been reported in at least 120 mammalian species across most major orders, including marsupials (in captivity) [1]. Alloparenting has been hypothesized to result from (1) kin selection [2], (2) reciprocal altruism [3], (3) improved parental experience and/or (4) misdirected care [1,4], but allonursing in particular appears to evolve where costs are low [5]. Kin selection involves related females sharing parental duties, including the nursing of each other’s young, as often occurs in communally breeding rodents [6] and carnivores [7] but it has also been invoked for adoption in an asocial sciurid [8]. Reciprocal altruism involves unrelated females sharing parental duties: young could gain immunological benefits such as antibodies from allosuckling, potentially increasing their survival [9], but there are no published cases of reciprocal allonursing among unrelated mammals in the wild [4]. Improved parental experience could occur when inexperienced individuals take on parenting duties, potentially increasing future reproductive success [10]; most studies, however, report that the more experienced females adopt following the loss of an offspring [4]. Misdirected care in the absence of kin benefits occurs in situations where females breed at high density and cannot avoid allonursing, such as in some bats and seals [1,11], or in species with poor offspring recognition, where juveniles may be confused and adopted following a disturbance [12].

Occurrences of adoption of pouch young in wild eastern grey kangaroos (*Macropus giganteus*) led us to investigate the factors responsible for this behaviour. Here we describe behavioural associations between mothers and adopted young in a high-density population of kangaroos and assess the validity of the adoption hypotheses listed above. Eastern grey kangaroos are gregarious herbivores, which live in fission-fusion groups [13]. Adult females do not form close associations with each other [14]. Females usually give birth to a single altricial offspring after a gestation of 36 days [15]. The young develops in the pouch and permanently emerges at about 10.5 months of age, following an ‘in-and-out’ period of about 5 weeks, with brief initial sorties from the pouch at 6.5 months of age [15]. Following permanent emergence from the pouch, the young is referred to as a young-at-foot and continues to suckle until at least 18 months of age [15]. Reproduction is seasonal, with most young permanently leaving the pouch in October and November in southeastern Australia [16,17].

## Materials and Methods

We sampled and measured 326 juvenile and 194 adult female eastern grey kangaroos in Wilsons Promontory National Park, Victoria, Australia, between 2008 and 2013. We marked all captured adults and 316 young in the pouch or at foot [18] and aged them according to body measurements [19]. We classified adult females according to incisor wear as prime-aged or old [20]. Adult females that were both small (arm length < 207 cm) and light (mass < 23.5 kg) at first capture were classified as 3-year-olds (young). We evaluated parity based on appearance of the pouch and teats [15]. We estimated body condition as the standardized residual of a linear regression of the logarithm of body mass on hind leg length [21], using different regressions for adult females, male pouch young and female pouch young. We collected tissue samples for genetic analyses from the ear of females and young. We estimated the density of females with large pouch young each winter by multiplying the estimated density of kangaroos in July from distance sampling along fixed transects[22] by the proportion of marked females with large pouch young in August/September. Densities of kangaroos ranged between 5 and 7/ha [23] and we marked approximately one half the adult females present each year. Only about 50% of marked females successfully raised offspring to the large pouch young stage (approximately 8 months of age) in any year (S1 Table). We assessed juvenile survival to 21 months of age. The main predator was the introduced red fox (*Vulpes vulpes*), which can kill young eastern grey kangaroos [24].

**Fig 1:**
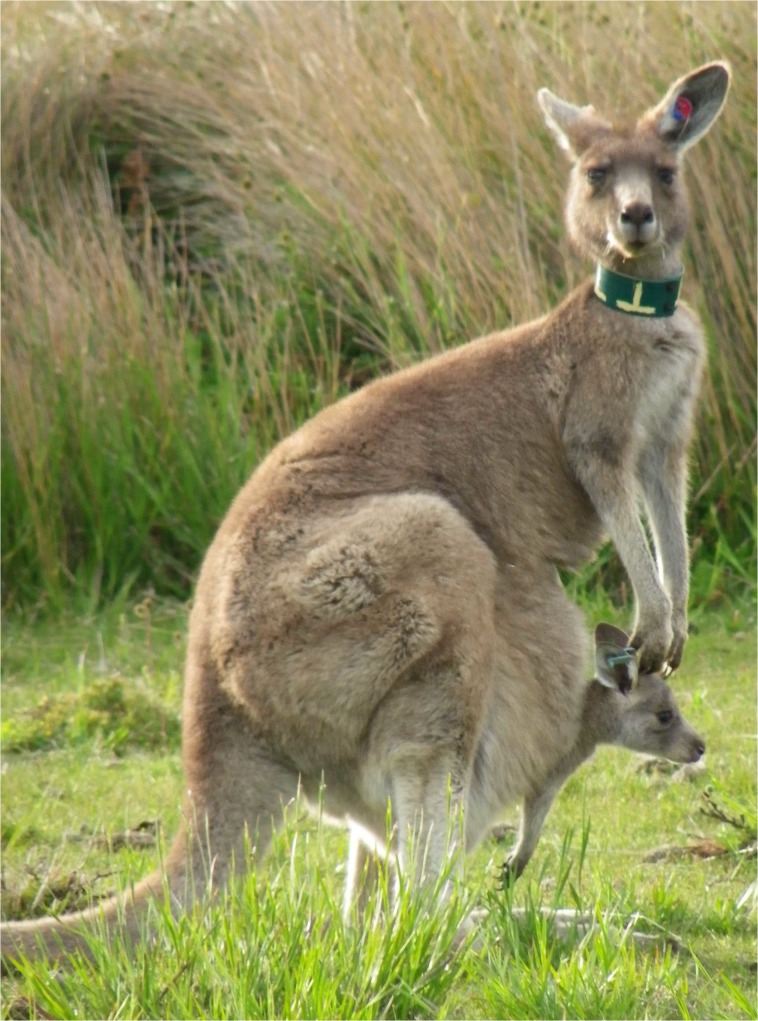
Female #81 with adopted young #531 in her pouch. The light blue eartag in the ear of #531 was applied when captured in the pouch of female #363. Photo courtesy of C. Le Gall-Payne.

We initially detected adoptions through observations of associations, pouch occupation and nursing between marked mothers and young (see Fig 1). To assess the effects of maternal age, body condition and population density on the likelihood of adoption and juvenile survival, we compared those factors for the 11 adoptive females for the year they adopted to all other years they were monitored (range 2–5 years). We fitted generalised linear mixed-effect logistic regressions with binomial errors in R version 2.15.2 [25], using maternal identity as a random factor. We sequentially removed the least significant parameter (based on its *P*-value, threshold ≥0.05) from models using stepwise backward selection [26]. Predictors for whether females adopted included age class, body condition, winter density of females with large pouch young and all 2-way interactions. Predictors for juvenile survival also included whether the offspring had been adopted. We did not include parity in analyses because it was correlated with age (all primiparous females were young) and was unknown at first capture. We compared the proportion of adoptive females that successfully raised an offspring to the large pouch young stage in the year following an adoption to the population average using a Fisher Exact Test. Intensive observations (approximately 45 h/month) from April 2010 to June 2012 allowed assessment of mother-offspring associations using half-weight indices (HWIs) calculated in SOCPROG 2.4 [27]. HWIs measure the proportion of time individuals are seen together in the same group [27] and we defined a group using the 10-m chain rule [28]. We compared 4-month HWIs between mothers and adopted young to those between mothers and biological offspring using *t*-tests. We also calculated HWIs between adoptive and biological mothers (if both individuals were known) from intensive observations taken in August through November 2010–2013. We investigated genetic relationships using 9 highly polymorphic microsatellite markers (G12-6, G16-1, G16-2, G19-1, G26-4, G31-1, G31-3, T3-1T and T32-1) [29,30], according to the methods of King et al. [31] and calculated pairwise relatedness (*r*) using KINGROUP v2 [32]. Mothers and putative young with mismatched genotypes without behavioural data on adoption were re-sampled and re-analysed for verification of non-relatedness. Spreadsheets of (1) characteristics of adoptive mothers and offspring survival for all years monitored, (2) genotypes of adoptive mothers and offspring and (3) HWIs of mothers with offspring are available at Dryad Digital Depository, http://dx.doi.org/10.5061/dryad.jr531.

The study was conducted under permits from the Victorian Department of Sustainability and Environment (#1004582, #1005558 and #100007062) and approved by the Animal Ethics Committees of the University of Melbourne (#0810628.1, #0911512.1 and #1312902.1) and the University of Queensland (#SIB/206/09/(NF)).

## Results

**Fig 2:**
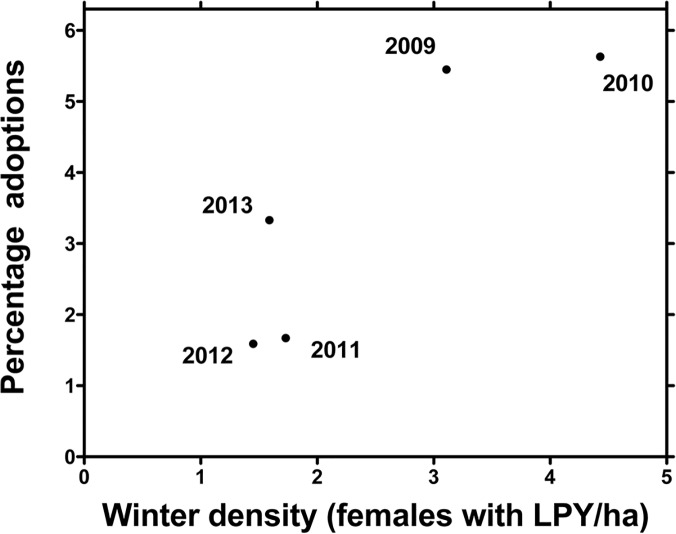
Annual percentage adoptions as a function of density of female eastern grey kangaroos with large pouch young (LPY) in winter at Wilsons Promontory National Park, Australia. Density was not estimated in 2008 before the study started.

We recorded 11 adoptions among a total of 326 juveniles (3%; Table 1). We detected eight adoptions through observations of associations and nursing between marked mothers and young, and another three through DNA analyses (Table 1). Four adoptions involved reciprocal switches between pairs of mothers (#6 with #115 and #64 with #310; Table 2) and four were by mothers whose own pouch young disappeared (#81, #166, #441 and #492; Table 2), of which three had been marked (those of #166, #441 and #492). One mother was seen to reject her offspring after a non-offspring had occupied her pouch, by kicking at it when it approached and attempted to follow her (#81). One mother was captured with two young in her pouch and subsequently abandoned her own offspring (#492; Table 1). Both sexes were adopted and adoptees varied in body condition (mean ± SE: −0.011 ± 0.017; Table 1). Young were adopted when 7–11 months of age and those that survived to permanent pouch emergence were allonursed on average for 6.1 months following adoption (Table 1). All adoptive mothers were already lactating and usually adopted young of the same age and sex as their biological offspring (Table 2). When the difference in estimated age of the two offspring was more than one week, the older and larger offspring was adopted (Table 2). Most adoptive mothers were prime-aged, multiparous and in above-average body condition when captured with a large pouch young, the time when adoptions occurred (Table 2), but the only significant parameter retained in the logistic regression model of the likelihood of adoption was density of females with large pouch young (Table 3; see Fig 2). Most adopted young survived to weaning at around 18 months (Table 1) and 6/11 adoptees survived to at least 59 months of age, including the four involved in reciprocal switches. Adoption did not improve juvenile survival (Table 4). Rather, survival to 21 months increased with maternal body condition and density of females with large pouch young (Table 4). Adopting did not reduce future reproduction because 82% of 11 females produced large pouch young the following year (Table 2) compared to the population average of 53% for females successfully raising offspring to large pouch young stage (Fisher Exact Test, *P* = 0.36). Twelve young were orphaned when 12–17 months of age during our 6-year study. None was adopted before or allonursed after permanent pouch exit, and adult females were generally aggressive towards them.

**Table 1:** Characteristics of adopted pouch young (PY) eastern grey kangaroos at Wilsons Promontory National Park, Australia, 2008–2013.

ID	Sex	Cohort	*r* with Mother A	*r* with Mother B (no. mismatched loci)	Type evidence^1^	ID PY replaced	Body condition	Age when detected (months)	Age last suckling (months)
89	female	2009	0.35	–0.08 (4)	pouch	116	–0.058	11.2	N/A
116	female	2009	0.44	0.05 (4)	assoc.	89	–0.021	11.0	16.8
132	male	2009	N/A	–0.11 (3)	DNA	N/A	0.009	9.2	N/A
202	male	2010	0.47	0.26 (2)	pouch	307	–0.026	10.9	16.1
307	male	2010	0.60	0.08 (3)	assoc.	202	N/A	11.0	15.2
321	male	2010	N/A	–0.22 (5)	pouch	239^2^	N/A	10.2	18.6
346	male	2010	N/A	–0.32 (7)	DNA	N/A	N/A	15.4^3^	21.4
466	female	2011	N/A	–0.10 (4)	DNA	unmarked	0.094	8.4	8.9^4^
531	male	2012	0.45	0.17 (2)	pouch	unmarked	–0.029	8.6	15.7^5^
615	female	2013	0.50	–0.12 (4)	pouch	622^2^	–0.052	9.9	10.7^4^
620	male	2013	N/A	0.08 (3)	pouch	584^6^	–0.003	7.3^7^	9.6^4^

Mother A is the putative biological mother and Mother B is the adoptive mother. ‘N/A’ refers to not available

^1^ pouch = seen in a second pouch; assoc. = seen suckling a second mother; DNA = seen only with one mother but genotype of young did not match mother.

^2^ Subsequently disappeared and presumably died.

^3^ Not individually recognisable until marked at 15.4 months of age.

^4^ Disappeared at this age and presumably died.

^5^ Disappeared at this age and was later found dead.

^6^ Abandoned soon after capture.

^7^ Found in the pouch with another young (#584) that was alone in the pouch when first captured at 1.4 months of age.

**Table 2:** Characteristics of adoptive female eastern grey kangaroos at Wilsons Promontory National Park, Australia, 2008–2013.

ID	Sex adopted	Cohort	ID other mother	Sex replaced	Age difference in young (d)	Body condition	Reproductive status	Reproductive success following year
115	female	2009	6	same	+6	+0.064	N/A	weaned son
6	female	2009	115	same	–6	+0.015	multiparous	weaned daughter
131	male	2009	N/A	N/A	N/A	+0.086	N/A	produced daughter^1^
310	male	2010	64	same	–1	+0.024	N/A	weaned daughter
64	male	2010	310	same	+1	+0.027	multiparous	weaned son
166	male	2010	N/A	same	–2	+0.046	multiparous	weaned daughter
303	male	2010	N/A	N/A	N/A	+0.022	N/A	lost small pouch young
349	female	2011	N/A	different	+13	+0.010	primiparous	weaned daughter
81	male	2012	363	same	N/A	N/A	multiparous	lost small pouch young
441	female	2013	36	same	+26	–0.027	multiparous	produced daughter^2^
492	male	2013	N/A	same	+9	–0.019	N/A	lost large pouch son

‘N/A’ refers to not available.

^1^ Disappeared at 17.1 months of age and presumably died.

^2^ Still alive as a young-at-foot at 12 months of age in January 2015.

**Table 3:** Parameter estimates for fixed effects retained in the generalised linear mixed-effects logistic regression model of whether or not 11 adult female eastern grey kangaroos adopted a pouch young at Wilsons Promontory National Park, Australia, 2009–2013 (*n* = 43).

	Estimate	SE	*z*	*P*
Intercept	–3.37	0.99	–3.41	0.001
Density of females with LPY^1^	0.84	0.32	2.61	0.009

^1^ large pouch young

**Table 4:** Parameter estimates for fixed effects retained in the generalised linear mixed-effects logistic regression model of offspring survival to 21 months for 11 adult female eastern grey kangaroos at Wilsons Promontory National Park, Australia, 2009–2013 (*n* = 43).

	Estimate	SE	*z*	*P*
Intercept	–6.46	2.38	–2.72	0.006
Maternal body condition	305.4	122.0	2.50	0.012
Density of females with LPY^1^	2.23	0.88	2.52	0.012
Maternal body condition X Density of females with LPY^1^	-88.8	37.4	-2.37	0.018

^1^ large pouch young

Mean pairwise relatedness *r* between 321 mothers and biological offspring was 0.48 ± 0.01 and pairs matched at all 9 loci. Adoptive mothers and young were not closely related (mean *r* = − 0.03 ± 0.05), with mismatches at 2–7 of 9 loci (Table 1). When genotypes of both biological and adoptive mothers were known (*n* = 6), they were not closely related (mean *r* = 0.03 ± 0.02). The six pairs of adoptive mothers and young studied in 2010–2012 associated as closely as did biological mother-young pairs. Adopted young showed a non-significant trend for greater association indices at 14–17 months compared to biological pairs (sons: mean HWI = 0.80 ± 0.07, *n* = 4 *vs*. 0.55 ± 0.04, *n* = 53 *t*_55_ = 1.85, *P* = 0.07; daughters: mean HWI = 0.87 ± 0.13, *n* = 2 *vs*. 0.72 ± 0.02, *n* = 33 *t*_33_ = 1.46, *P* = 0.15). No differences occurred at 18–21 months (*t* < 0.67, *P* > 0.50) or 22–25 months (*t* < 0.69, *P* > 0.49). The three known pairs of adoptive and biological mothers did not associate closely in August–November, when adoptions occurred (mean HWI = 0.07 ± 0.06).

## Discussion

Marsupial reproduction, with extended lactation of young in the pouch, has long been exploited in captivity for experimental manipulation and cross-fostering, especially for conservation of rare macropodids [33,34]. Reproductive females readily accept non-offspring pouch young of roughly the same age as that removed, even from other species [34,35]. However, our observations of pouch young adoption are the first in wild kangaroos.

Kin selection does not explain adoptions in kangaroos because females did not adopt closely related young; *r*-values between mothers and adopted young were on average close to zero. Communal rearing of mammalian young by related mothers seems to be key for allonursing to be maintained by kin selection [4] but kinship plays little role in the social structure of female eastern grey kangaroos [14].

Most reciprocal exchanges of parental care in unrelated mammals take the form of babysitting [1], but in eastern grey kangaroos the switch was permanent until weaning. We recorded two pairs of females that switched offspring, which is the first documented evidence for mutual cooperation involving reciprocal allonursing among unrelated wild mammals. Reciprocity theory requires equal input from both parents because altruism is open to cheating [36]. Where pouch young were switched, all four mothers exclusively nursed the adopted young for at least 6 months and ceased care of their biological offspring. The reward for mutual cooperation in switching pouch young could be improved offspring survival due to immunological benefits [9]. Unlike eutherian mammals, marsupials receive maternal antibodies over a prolonged period throughout pouch life [37], so adopted young could obtain additional immune compounds from their adoptive mother, increasing survival. However, we did not detect improved juvenile survival in adopted young. Additional observations are required to assess survival benefits of reciprocal switches. Both reciprocal switches that we recorded occurred in years of high density, when there were many mothers with large pouch young and mothers were in good body condition. It is thus possible that in-and-out pouch young are participating in a kind of musical chairs game: if a lactating mother leaves a group without being followed by her pouch young (and the young does soon not find her), the young needs to quickly find another lactating female with a vacant pouch, or it will die of exposure overnight. Dependent offspring that have not permanently emerged from the pouch follow their mothers closely and give distress calls when lost [38]. Because we had marked three of the four biological young that disappeared after their mother adopted another young, we would have detected if another female had adopted them. Thus only about one half of adoptions involved reciprocity. Also, since individual mothers spent very little time together, it is unlikely that adoptive females were monitoring the behaviour of females with their biological offspring.

Adoption was unlikely to improve parental experience because only one of the 11 adoptive mothers was young and primiparous. Although all other adoptive mothers were prime-aged and most were in above-average body condition, these variables did not affect the likelihood of adoption in eastern grey kangaroos. In contrast, adoptive northern elephant seal (*Mirounga angustirostris*) mothers were typically young and inexperienced [10].

The hypothesis of misdirected care to explain adoption is contingent upon poor mother-offspring recognition and/or there being little cost to adoption compared to the costs of detecting, recognising and rejecting non-offspring [4]. Kangaroos appear to recognise offspring through olfaction [38], as do most mammals [39]. Because known orphans were never adopted, we speculate that adoptions may have occurred during a disturbance, with females accepting non-offspring into the pouch without verifying their identity before fleeing. Normally, female kangaroos nose juveniles before allowing them to enter the pouch and are aggressive towards any non-offspring that approach [38]. Taking the time to identify offspring in a disturbance would elevate the risk of mother and young becoming separated, with potentially lethal effects on the young. Once inside the pouch, the adopted young may take on the female’s odour and be difficult to distinguish from biological offspring. As for the cost of allonursing from pouch emergence to weaning, adoptions occurred at a time when milk production would soon be declining [40] and adoptive females did not appear to have reduced reproduction the following year. Detection of a cost to reproduction is difficult, however, without manipulation of reproductive effort [41]. Adoption in kangaroos thus appeared to incur few costs once young were permanently emerged from the pouch, but when adoptions were not reciprocal (43% of cases), the adoptive mother’s own offspring died, indicating an unusually high cost [5]. That females were more likely to adopt in years with many females with large pouch young also supports the hypothesis of misdirected care, since accidental adoptions are more likely to occur under high-density breeding conditions [1]. Density positively affects the probability of allonursing in Hawaiian monk seals (*Monachus schauinslandi*), another species showing misdirected parental care [42].

Simultaneous adoption of a second offspring into the pouch in wild monotocous marsupials is extremely rare but has been reported in a high-density urban population of common brushtail possum (*Trichosurus vulpecula*) [43]. Eymann et al. [43] suggested that kin selection was responsible for this adoption because the individuals shared mitochondrial DNA haplotypes, but relatedness was not estimated. Biological twins have occasionally been observed in several macropodids, including eastern grey kangaroos, however one twin always dies in the pouch at a young age [15,44,45].

Robust examples of adoption are rare in wild mammals. A 0.2% frequency of adoption in red squirrels (*Tamiasciurus hudsonicus*) was ascribed to kin selection [8]. Adoption in our 6-year study of wild eastern grey kangaroos was an order of magnitude more frequent, at 3%. Since (1) adoptive kangaroo mothers were not closely related to each other or to the adoptees, (2) reciprocal switches did not always occur, (3) switch mothers did not associate closely, (4) most adoptive mothers were not young and inexperienced, (5) adoption did not improve offspring survival, (6) females were more likely to adopt at high density and (7) adoptive mothers and young associated as closely as did biological pairs, we conclude that adoption was likely caused by misdirected care. Our results suggest that mother-offspring recognition mechanisms may be poorly developed in eastern grey kangaroos. The relatively high frequency of adoptive behaviour observed in this population indicates that maladaptive behaviour with possible highly detrimental fitness consequences sometimes occurs in natural populations.

## Supporting Information

S1 Table Numbers of marked adult females raising an offspring to large pouch stage (approximately 8 months of age) each year from 2008 to 2013 at Wilsons Promontory National Park, Australia. (DOCX)
