# Supplementary material for: Adoption in Eastern Grey Kangaroos: A Consequence of Misdirected Care?
Source: PLoS One. 2015 May 13;10(5):e0125182. doi: 10.1371/journal.pone.0125182 (PMC4430339; doi:10.1371/journal.pone.0125182)
Supplement: S1 Table — (DOCX) [file pone.0125182.s001.docx]

**Table S1. Numbers of marked adult female kangaroos raising an offspring to large pouch stage (approximately 8 months of age) each year from 2008 to 2013 at Wilsons Promontory National Park, Australia.**

| Year | Successful | Unsuccessful | Total | Success rate (%) |
| --- | --- | --- | --- | --- |
| 2008 | 17 | 25 | 42 | 40 |
| 2009 | 59 | 16 | 75 | 79 |
| 2010 | 71 | 22 | 93 | 76 |
| 2011 | 56 | 76 | 132 | 42 |
| 2012 | 64 | 69 | 133 | 48 |
| 2013 | 62 | 81 | 143 | 43 |
| Total | 329 | 289 | 618 | 53 |
